# Mental Health Care in the African-American Community

**Published:** 2008-09-15

**Authors:** Jeane W Anastas

**Affiliations:** Silver School of Social Work, New York University, New York, New York

**Figure:**
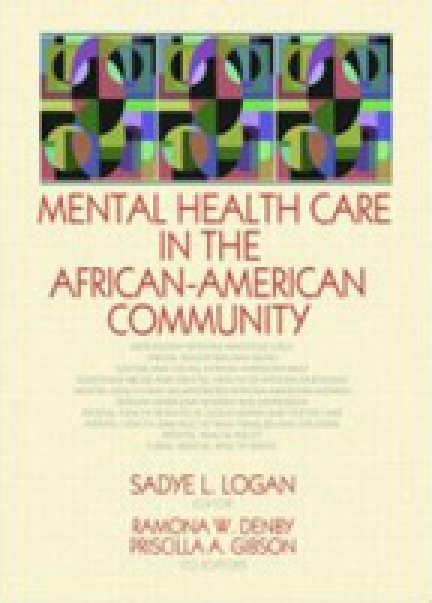


*Mental Health Care in the African-American Community* aims to educate providers of mental health services about the historical and contextual factors that influence the prevalence and treatment of mental health problems among African Americans in the United States. The book's life-course framework incorporates what is known in social science as the "person-in-environment perspective," in which developmental, cohort, family, resource, community, service system, and social policy factors must all be considered to prevent and effectively treat mental and physical illness. The premise of the book is the idea that there is a "serious mismatch" between how mental illnesses and substance abuse are generally understood and treated and the perceptions and needs of African American individuals, families, and communities. Its editors present ample evidence that this premise is correct.

The chapters are not organized around the more common (and often more limited) framework of cultural competence. Rather, after presenting historical, theoretical, and contextual critiques of the current situation, the editors devote most of the text to describing and analyzing a long list of specific mental health issues that affect different parts of the African American community over the lifespan. These chapters emphasize family, community, and social factors, especially the effects of oppression, that influence the emergence or persistence of these problems. Many chapters describe promising alternative service delivery models that have at least a beginning evidence base documenting their effectiveness. Each chapter concludes with classroom exercises and discussion points — often very creative ones — which suggests that the editors also intend the book to be used in social work and other programs in which mental health care providers, administrators, and policy makers are educated.

The book is broad in scope. Chapters about children and adolescents address limited special education services in schools and mental health services for children in the foster care system; stereotyped views of African American girls that contribute to their social problems; suicide among young African American men; and a rite-of-passage model aimed at improving mental health and self-esteem among young African American men. Issues for multiracial children and families and challenges faced by high-achieving African American young adults in predominantly white colleges are also discussed.

Chapters about adults and families address substance use and abuse; depression among women; mental health problems among incarcerated women and among women with HIV/AIDS; the utility of kinship care for promoting mental health in African American families; and mental health service needs in rural African American communities in the South.

The one chapter covering mental issues among elderly African Americans provides useful information on the economic and social circumstances of this age group, but the chapter has some gaps. The emerging issue of disparities in the early identification and treatment of dementia in African Americans, which is a serious problem now that the progression of this condition can be slowed, is not discussed. In addition, the community outreach model presented in the chapter is excellent but is not elder-specific. The book concludes with chapters on general social policy, the Afrocentric perspective, and thoughts for the future.

As an outsider to the African American community, I was aware of some of the mental health issues, theories, and interventions presented in this book. For example, I knew about many of the problems originating from recent drug policy in the United States — the late 20th-century "war on drugs" — but not about the much longer history of drug policies' differential effects on the urban African American community. Now I more strongly question current political rhetoric, some prevention and treatment programs, and social policies that address substance use and abuse in these communities. I suspect that most readers will gain valuable perspectives on a range of mental health issues from this book. The authors are to be commended for bringing a consciousness of the importance of gender  to their analysis of mental health problems, prevention, and treatment, and the systems of oppression that affect the African American community.

The perspective of this book on mental health in African American communities is holistic and not medical. Hence, much attention is given to the social, economic, and cultural factors that contribute to mental illness, family responses to those affected, and how mental health programs might treat them more effectively. At times the authors sacrifice depth in description of the epidemiology of specific mental health problems in this community. They also do not reference the language of health disparities, which is unfortunate because this construct seems to be gaining some political traction in the United States (however distant the reality of fair treatment may be). Rather, this volume challenges mental health care providers to think broadly about mental health issues in African American communities and to be creative in their efforts to prevent and ameliorate these problems in family, institutional, and community contexts.

